# Perioperative dynamics and significance of amino acid profiles in patients with cancer

**DOI:** 10.1186/s12967-015-0408-1

**Published:** 2015-01-27

**Authors:** Yu Gu, Tianxiang Chen, Suzhen Fu, Xin Sun, Lingyan Wang, Jian Wang, Yingfeng Lu, Songming Ding, Guodong Ruan, Lisong Teng, Min Wang

**Affiliations:** Department of Surgical Oncology, First Affiliated Hospital, College of Medicine, Zhejiang University, Hangzhou, China; Department of Thoracic Surgery, First Affiliated Hospital, College of Medicine, Zhejiang University, Hangzhou, China; State Key Laboratory for Diagnosis and Treatment of Infectious Diseases, Department of Infectious Diseases, First Affiliated Hospital, College of Medicine, Zhejiang University, Hangzhou, China; Division of Neurobiology, Department of Psychiatry and Behavioral Sciences, Johns Hopkins University School of Medicine, Baltimore, USA; Biomedical Research Center, Zhongshan Hospital, Fudan University, Shanghai, China; Division of Hepatobiliary and Pancreatic Surgery, Department of Surgery, First Affiliated Hospital, College of Medicine, Zhejiang University, Hangzhou, China; Department of Oncology, The Second Hospital of Shaoxing, Shaoxing, China

**Keywords:** Amino acid profile, Plasma, Metabolism, Cancer, Perioperation

## Abstract

**Background:**

Metabolome analysis including amino acid profile is under investigation as an approach in cancer screening. The present study aims to analyze plasma free amino acid (PFAA) profiles in cancer patients and investigate their potential as biomarkers of malignancy.

**Methods:**

Plasma samples from 56 gastric cancer patients, 28 breast cancer patients, 33 thyroid cancer patients, and 137 age-matched healthy controls were collected in the study. PFAA levels were measured and their perioperative alterations were analyzed. Biological effects of ten cancer-related amino acids were further validated in gastric and breast cancer cells.

**Results:**

We found that PFAA profiles of cancer patients differed significantly from those of healthy controls. Decreased concentrations of PFAAs were associated with lymph node metastases in gastric cancer. Levels of PFAAs such as aspartate and alanine increased after tumor resection. PFAA levels correlated with clinical tumor markers in gastric cancer patients and pathological immunohistochemistry markers in breast cancer patients. Specifically, alanine, arginine, aspartate and cysteine had proliferative effects on breast cancer cells. Proliferation of gastric cancer cells was promoted by cysteine, but inhibited by alanine and glutamic acid. Furthermore, alanine treatment decreased total and stable fraction of gastric cancer cells, and alanine and glutamic acid induced apoptosis of gastric cancer cells.

**Conclusions:**

PFAA patterns in cancer patients are altered perioperatively. Tumor-related amino acids identified by dynamic study of PFAA patterns may have the potential to be developed as novel biomarkers for diagnosis and prognosis of cancer patients.

**Electronic supplementary material:**

The online version of this article (doi:10.1186/s12967-015-0408-1) contains supplementary material, which is available to authorized users.

## Background

Cancer will be the leading cause of death within the next several decades, and the early detection of cancer is crucial for improved survival of patients [1]. Current cancer screening techniques such as endoscopy for gastric cancer (GC), mammography for breast cancer (BC), and ultrasound for thyroid cancer (TC) are important in clinical applications, although they are limited by invasiveness, radiation exposure and high cost [2-4]. Pre-existing biomarkers such as carcinoembryonic antigen (CEA) and carbohydrate antigen (e.g., CA 15–3 and CA19-9) are also frequently used to monitor tumor response, however, their sensitivities and specificities are still controversial for early detection [5,6]. Therefore, novel methods with noninvasiveness, reliable sensitivity and specificity are constantly desired for cancer screening.

Amino acids have been considered as potential targets due to their roles as metabolites and metabolic regulators. Recent studies have highlighted the diagnostic and the prognostic potential of amino acids in a range of human diseases such as schizophrenia, chronic obstructive pulmonary disease, and diabetes [7-9]. Cancer is viewed as a metabolic disease with an enhanced metabolism, since malignant cells require more amino acids to synthesize proteins and nucleic acids [10,11]. Circulating amino acids exhibited by plasma free amino acids (PFAAs) may represent tumor-induced protein metabolism in patients with malignancy [12]. Patients with cancer had PFAA alterations dependent upon their cancer types [13-17]. PFAA profiles frequently correlated with the organ-site origin among different cancers [18]. It is possible to monitor and evaluate cancer patients before and after treatment using a snapshot of amino acid metabolisms present at time points [19]. However, there is limited information on PFAA profiles of cancer patients during the perioperative period and the influence of tumor removal on general amino acid metabolisms is still largely unknown.

In the present study, we investigated the variation of amino acids profiles between cancer patients and healthy controls (HCs), by examining peripheral blood metabolites from 56 GC patients, 28 BC patients, 33 TC patients, and 137 age-matched HCs. Tumor-specific PFAA profiles were identified. Moreover, paired plasma samples were collected from 15 GC patients and 10 BC patients before intervention and 5–15 days after tumor resection, and the perioperative PFAA levels were analyzed to reveal the effect of tumor burden on PFAA profiles. Cytological effects of tumor-related PFAAs on gastric and breast cancer cells were further examined *in vitro*. The present study demonstrates that dynamic observations of PFAA profiles in cancer patients may provide an insight into cancer metabolism and may be an alternative to detect tumors.

## Methods

### Subjects

Data from 117 patients with GC, BC and TC were analyzed in the present study. The diagnoses of the primary cancer were histologically made at the Cancer Center of the First Affiliated Hospital, College of Medicine, Zhejiang University, Hangzhou, China. One hundred and thirty-seven HCs were recruited and screened for serum levels of tumor markers, including carcinoembryonic antigen (CEA), carbohydrate antigen19-9 (CA19-9), carbohydrate antigen 125 (CA125), carbohydrate antigen 15–3 (CA15-3) and alpha-fetoprotein (AFP). The present study was conducted according to the Declaration of Helsinki and all procedures involving human subjects were approved by the Medical Ethics Committee in the First Affiliated Hospital, College of Medicine, Zhejiang University. Verbal informed consent was obtained from all subjects, witnessed, and formally recorded. Stages of GC, BC, and TC were determined according to Tumor–Node–Metastasis (TNM) Classification of Malignant Tumors from the Seventh Edition of the American Joint Committee on Cancer [20]. Clinical information listed in Additional file 1 and Additional file 2 was obtained from clinical records.

### Cell culture

Human breast cancer cell lines MDA-MB-231 (Cat. No. HTB-26), MCF7 (Cat. No. HTB-22) and BT-474 (Cat. No. HTB-20) and human gastric cancer cell line AGS (Cat. No. CRL-1739) were obtained from American Type Culture Collection (Manassas, VA, USA). Human gastric cancer cell line SGC-7901 was obtained from the Cell Bank of Type Culture Collection of the Chinese Academy of Sciences (Shanghai, China) and human gastric cancer cell line MKN45 was obtained from the Chinese Academy of Medical Sciences Cancer Institute (Beijing, China). Cells were cultured at 37°C in the presence of 5% CO_2_ in RPMI 1640 medium (Gibco BRL, Carlsbad, CA, USA) supplemented with 10% fetal bovine serum (HyClone, Logan, UT, USA).

### PFAA analyses

**Figure 1:**
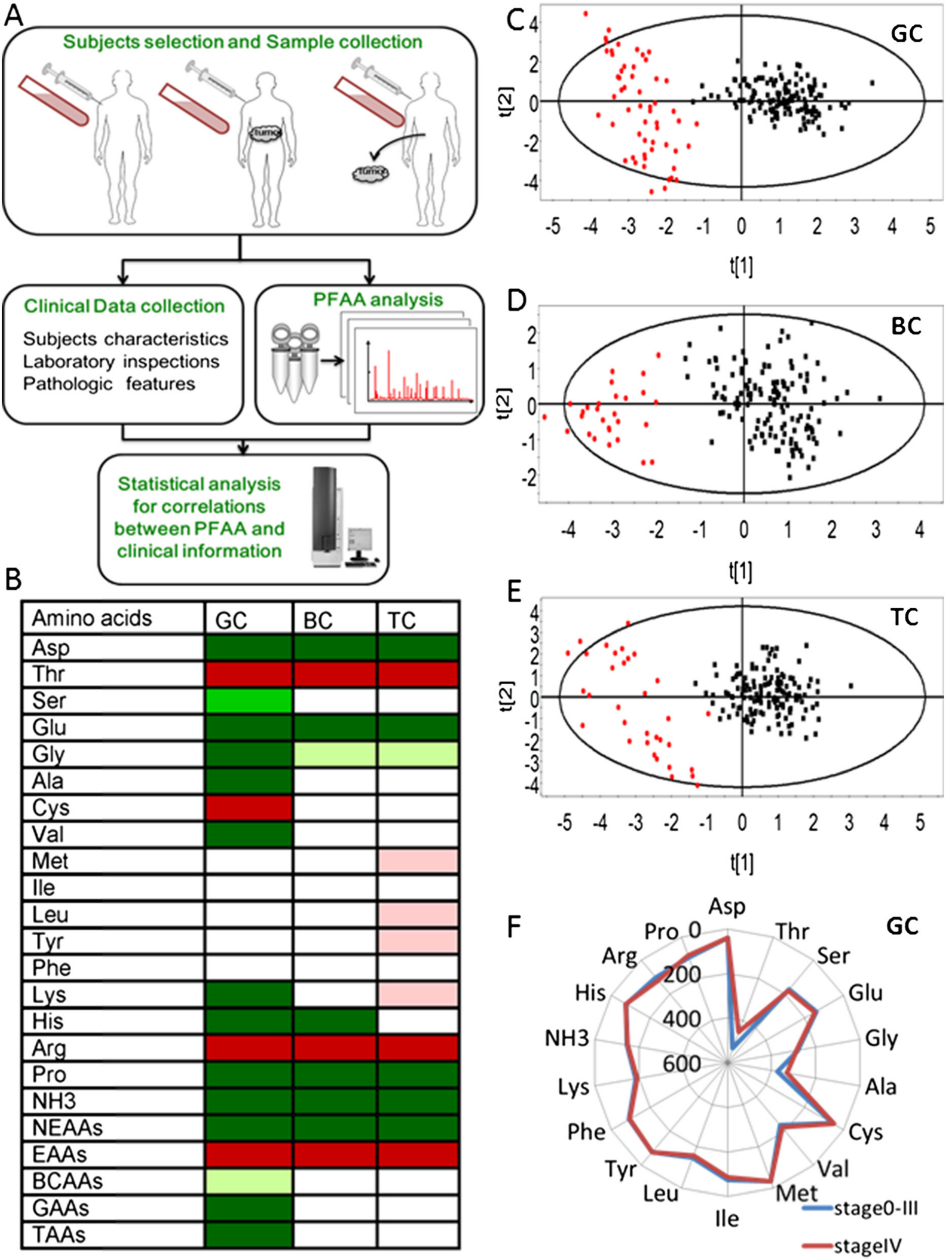
**PFAA profiling of patients with cancers.** Schematic procedures of PFAA screening **(A)** and PFAA profile alterations in 117 cancer patients including 56 GC, 28 BC and 33 TC, compared to 137 HCs **(B)** are presented. Colored cells indicated levels of amino acids increased in cancer patients at p < 0.001 (dark red), p < 0.05 (pink), decreased in cancer patients at p < 0.001 (dark green), p < 0.01(green) and p < 0.05 (light green), respectively. PLS-DA scores plots showed a separation between control subjects (black) and cancer patients (red) in GC group **(C)**, BC group **(D)** and TC group **(E)**. For GC group, R^2^ = 0.852, Q^2^ = 0.837; for BC group, R^2^ = 0.786, Q^2^ = 0.727; for TC group, R^2^ = 0.792, Q^2^ = 0.761. Altered amino acid profiles of GC patients at early and advanced tumor stages are presented **(F)**. The axes show the concentrations (μM) of each amino acid for discriminating relative early stage from advanced stage. Blue line stands for PFAAs of GC patients at stage 0-III, red line for PFAA of GC patients at stage IV. NEAAs: non-essential amino acids, EAAs: essential amino acids, BCAAs: branched-chain amino acids, GAAs: glucogenic amino acids, TAAs: total amino acids, GC: gastric cancer, BC: breast cancer, TC: thyroid cancer, HC: healthy control.

Blood was collected from veins immediately after admission before the intervention and 5–15 days after tumor resection in patients with cancer who had an overnight fast. Blood was placed in vacuum tubes with EDTA anticoagulant (BD Biosciences, San Diego, CA, USA), and centrifuged at 1,000 × g for 10 min. Plasma was stored at −80°C prior to use. Plasma concentrations of amino acids were measured by Hitachi amino acid analyzer L-8800 (Hitachi High-Tech, Tokyo, Japan) with colorimetric analysis as previously described [21]. Briefly, the cryopreserved plasma at 0.5 ml was added and mixed with 4% sulfosalicylic acid at 1.5 ml, and then centrifuged at 26,900 × g for 15 min. The supernatant at 0.02 ml was analyzed by the amino acid analyzer with spectrophotometrical detection after postcolumn reaction with ninhydrin reagent. The flushing fluid was 0.2 mM citric acid buffer at pH 3.3 and standard products and test kits were provided by the manufacturer. The ammonia is measured using an automatic biochemical analyzer Beckman CX9 (Beckman, Brea, CA, USA) with enzymatic determination, according to the manufacturer’s introductions. The amino acids and related molecules (18 compounds) measured in the analysis included alanine (Ala), arginine (Arg), aspartate (Asp), cysteine (Cys), glutamic acid (Glu), glycine (Gly), histidine (His), isoleucine (Ile), leucine (Leu), lysine (Lys), methionine (Met), ammonia (NH_3_), phenylalanine (Phe), proline (Pro), serine (Ser), threonine (Thr), tyrosine (Tyr), and valine (Val). The plasma levels of amino acids were expressed in μM. Procedures of PFAA profiling were shown in Figure 1A.

### Cell proliferation assay

Cell proliferation was measured by Cell Counting Kit-8 (CCK-8) detection kit (Dojindo, Kumamoto, Japan). Cells were plated in 96-well plates at a concentration of 5 × 10^3^ cells per well and incubated with Ala, Arg, Asp, Cys, Glu, Gly, His, Ser, Thr, or Val at 10 mM for 24, 48, or 72 h, respectively. The amino acids were purchased from Sigma-Aldrich (St. Louis, MO, USA). After treatment, 10 μl of CCK-8 per well was added and the cells were incubated at 37°C for 1 h. Proliferation was determined by absorbance at 450 nm using ELx800 Absorbance Microplate Reader (Biotek, Winooski, VT, USA). All experiments were repeated at least three times.

### Dynamic monitoring of cell biobehaviors

Cells were plated into 24-well plates with a density of 1 × 10^4^ cells per well, challenged with 10 mM Ala or not, and imaged on Cell-IQ cell culturing platform (Chip-Man Technologies, Tampere, Finland) every 5 min for 72 h. Cell-IQ system uses machine vision technology to monitor and record time-lapse data. It can also quantify cell functions and morphological parameters, automatically discriminate the dividing and stable cell stages, and calculate the total cell numbers during proliferation [22,23]. Differentiated cell is at the dividing cell stage and appears round, and bright while cell is at the stable cell stage and appears spindle and dark in Cell-IQ system. Four visual fields per well were automatically selected by the system. Total cell numbers and numbers of stable and differentiated cells were analyzed by a freely distributed Image software (Cell-IQ Imagen v2.9.5c, McMaster Biophotonics Facility, Hamilton, Canada), using the Manual Tracking plug-in created by Fabrice Cordelieres (Institut Curie, Orsay, France).

### Flow cytometry

Cell apoptosis was detected using the Annexin V-fluorescein isothiocyanate (FITC)/propidium iodide (PI) apoptosis detection kit (BD Biosciences) according to the manufacturer’s instructions. After treated with 10 mM Ala or Glu for 48 h, cells (1 × 10^6^/well) were collected, centrifuged, and resuspended in 500 μl of 1 × binding buffer. Annexin V-FITC (5 μl) and PI (5 μl) were then added to each tube. The tubes were incubated in the dark at room temperature for 20 min. Immediately after incubation, cell apoptosis was assessed on a flow cytometry (BD Biosciences). Representative images of experiments were shown. All experiments were repeated at least three times.

### Measurement of mitochondrial membrane potential

Mitochondrial membrane potential (MMP) was measured using a fluorescent, lipophilic and cationic probe JC-1 (5,5′,6,6′-tetrachloro-1,1′,3,3′-tetraethylbenzimidazolcarbocyanineiodide) (Beyotime Biotech, Nantong, China) according to the guideline from the manufacturer. Briefly, after indicated treatments, cells were incubated with JC-1 staining solution (5 μg/ml) for 20 min at 37°C and then rinsed twice with PBS. The fluorescence intensity of JC-1 and its cellular distribution were imaged under a Leica DMIRE2 confocal fluorescence microscope (Leica Microsystems AG, Wetzlar, Germany) equipped with Leica Confocal Software v.2.61. At least 6 visual fields in each were examined by 2 independent investigators. Representative images of experiments were shown. All experiments were repeated at least three times.

### Statistics

Differences among groups were analyzed by Student’s *t*-test or one-way ANOVA, as appropriate. Paired-sample *t*-test was applied to analyze differences of amino acid concentrations of patients before and after tumor resection. Correlations between clinical characteristics and amino acid concentrations were performed by Spearman’s rho test and Pearson’s test, as appropriate. A two-tailed value of p < 0.05 was considered statistically significant. Statistical analyses were performed using SPSS 18.0 software (SPSS Inc., Chicago, IL, USA). Following data normalization, the data set was input into SIMCA-P 11 software (Umetrics Inc., Umea, Sweden), partial least squares-discriminant analysis (PLS-DA) was used to discriminate between cancer patients and controls [16].

## Results

### Characteristics of subjects

The data sets comprised of 293 blood samples from 117 cancer patients, including 56 with GC, 28 with BC, 33 with TC, and 137 age- and gender-matched HCs. There was no significant loss of patient body weight or significantly decreased in serum albumin level before admission to avoid potential influence of malnutrition-associated metabolic changes (Additional file 1). The numbers of patients at each stage according to the type of cancer: were subdivided as 16/56 (28.6%) at stages 0 and I, 5/56 (8.9%) at stage II, 19/56 (33.9%) at stage III, and 12/56 (21.4%) at stage IV in GC patients; 7/28 (25%) at stage I, 18/28 (64.3%) at stage II, 3/28 (10.7%) at stage III, and 0/28 at stage IV in BC patients; 27/33 (81.8%) at stages 0 and I, 0/33 at stage II, 3/33 (9.1%) at stage III and 2/33 (6.1%) at stage IV in TC patients, as shown in Table 1.

**Table 1:** **Clinical characteristics of subjects**

**Characteristic**		**Number**		
		**BC**	**GC**	**TC**
**Patients**		28	56	33
**Sex**	F/M	26/2	19/37	22/11
**Age**	Mean (yrs)	57.5	61.9	45.0
**Tumor size**	<=5 cm	26	29	32
	>5 cm	2	23	1
**Primary tumor**	T0-3	28	24	33
	T4	0	28	0
**Lymph nodes metastasis MMEMmetastasis**	Negative	15	21	19
	Positive	13	31	14
**Distant metastasis**	Negative	28	40	33
	Positive	0	12	0
**Stages** ^**#**^	0	0	2	0
	I	7	14	27
	II	18	5	0
	III	3	19	3
	IV	0	12	2
	Uncharacterized	0	4	1

^#^Cancer stages were determined according to the International Union Against Cancer TNM Classification of Malignant Tumors, 7th edition.

### Difference of PFAA profiles between healthy controls and patients with cancers

Alteration patterns in concentrations of PFAA in 117 cancer patients and 137 HCs were listed in Figure 1B. PFAA profiles of patients with three types of cancers were significantly different from those of HCs. Cancer patients had significantly higher levels of Thr, Arg, and essential amino acids (EAAs), and significantly lower levels of Asp, Glu, Gly, Pro, non-essential amino acids (NEAAs), and NH3, as compared to HCs (p < 0.05 or less, respectively, Figure 1B and Additional file 3). Using PLS-DA we found separation between groups of cancer patients and controls. The scores plot shows each set of two groups scattering into different regions, representing a good separation of GC, BC or TC group from HC group with corresponding plasma amino acid patterns (Figure 1C-E). The variation of plasma amino acid profile at different disease stages is considered to be important [12]. Most of BC and TC patients in our study were categorized as early stage and only a few were defined as advanced stage, as shown in Table 1. In GC group, 12 cases were categorized as advanced stage (stage IV), however, their PFAAs were not significantly different from those at relative early stage (stage 0-III) (Figure 1F and Additional file 4). When we excluded data from GC patients at stage IV, PFAA profile in GC patients at relative early stage (stage 0-III) is still different from that in HCs (Additional file 5).

### Difference between types of cancers

Disease-specific alterations of amino acid profiles were further explored by analyzing PFAA profile difference of each cancer patients and HCs (Figure 1B). Level of His was significantly lower in patients with BC. Levels of Ser, Ala, Val, Lys, His, branched-chain amino acids (BCAAs), glucogenic amino acids (GAAs), and total amino acids (TAAs) were significantly lower in patients with GC. Patients with TC had significantly higher levels of Met, Leu, Tyr, and Lys. PFAA alteration patterns of patients with each type of cancer were different from each other as shown in Additional file 3 and Additional file 5.

### Association of PFAA profile with GC clinicopathological characteristics

The association of plasma amino acid profiles with cancer clinicopathological characteristics, which includes tumor size, invasiveness and metastasis, was further validated in the present study. Because most of BC and TC patients were at relative early stages, we focused on the GC group which is composed of both early and late stage cases. By comparing positive lymph node metastases group versus negative node metastases group, we found that the concentrations of Thr, His, EAAs, and GAAs were decreased (p < 0.05) (Table 2). Moreover, the decrease in Thr level was significantly associated with larger tumor size (>5 cm) and deeper invasion (T ≥ 4) (p < 0.05) (Additional file 6).

**Table 2:** **Association of plasma free amino acid profiles with lymph nodes metastasis status in gastric cancer patients**

	**Lymph node metastasis**		
	**Negative**	**Positive**	**P-value**
**Asp**	40.43 ± 8.84	39.65 ± 15.42	0.84
**Thr**	573.42 ± 151.47	482.93 ± 133.95	0.03*
**Ser**	184.48 ± 51.23	166.08 ± 54.06	0.22
**Glu**	143.20 ± 44.02	142.54 ± 51.59	0.96
**Gly**	314.26 ± 108.72	261.57 ± 85.22	0.06
**Ala**	402.78 ± 144.67	338.41 ± 115.88	0.08
**Cys**	57.73 ± 16.46	55.65 ± 19.27	0.69
**Val**	243.34 ± 59.76	223.94 ± 51.90	0.22
**Met**	34.85 ± 9.21	31.90 ± 10.38	0.30
**Ile**	77.43 ± 16.51	78.43 ± 26.45	0.88
**Leu**	152.75 ± 36.54	145.56 ± 37.56	0.50
**Tyr**	77.68 ± 33.21	75.80 ± 34.56	0.85
**Phe**	99.41 ± 22.06	90.22 ± 24.55	0.17
**Lys**	199.46 ± 48.14	175.67 ± 51.18	0.10
**His**	84.32 ± 22.81	71.23 ± 15.60	0.02*
**Arg**	113.70 ± 31.05	101.86 ± 40.30	0.26
**Pro**	96.68 ± 32.29	95.43 ± 41.45	0.91
**NH3**	139.91 ± 61.67	148.00 ± 60.06	0.64
**NEAAs**	1419.42 ± 328.68	1253.62 ± 355.51	0.10
**EAAs**	1380.65 ± 280.45	1213.08 ± 298.31	0.04*
**BCAAs**	473.52 ± 106.55	447.93 ± 107.62	0.40
**GAAs**	2213.05 ± 483.57	1923.79 ± 486.47	0.04*
**TAAs**	2939.98 ± 613.49	2614.70 ± 640.08	0.07

The plasma levels of amino acids were expressed in μM.

*Stands for p-values less than 0.05.

### Perioperative alterations of PFAA profiles

**Figure 2:**
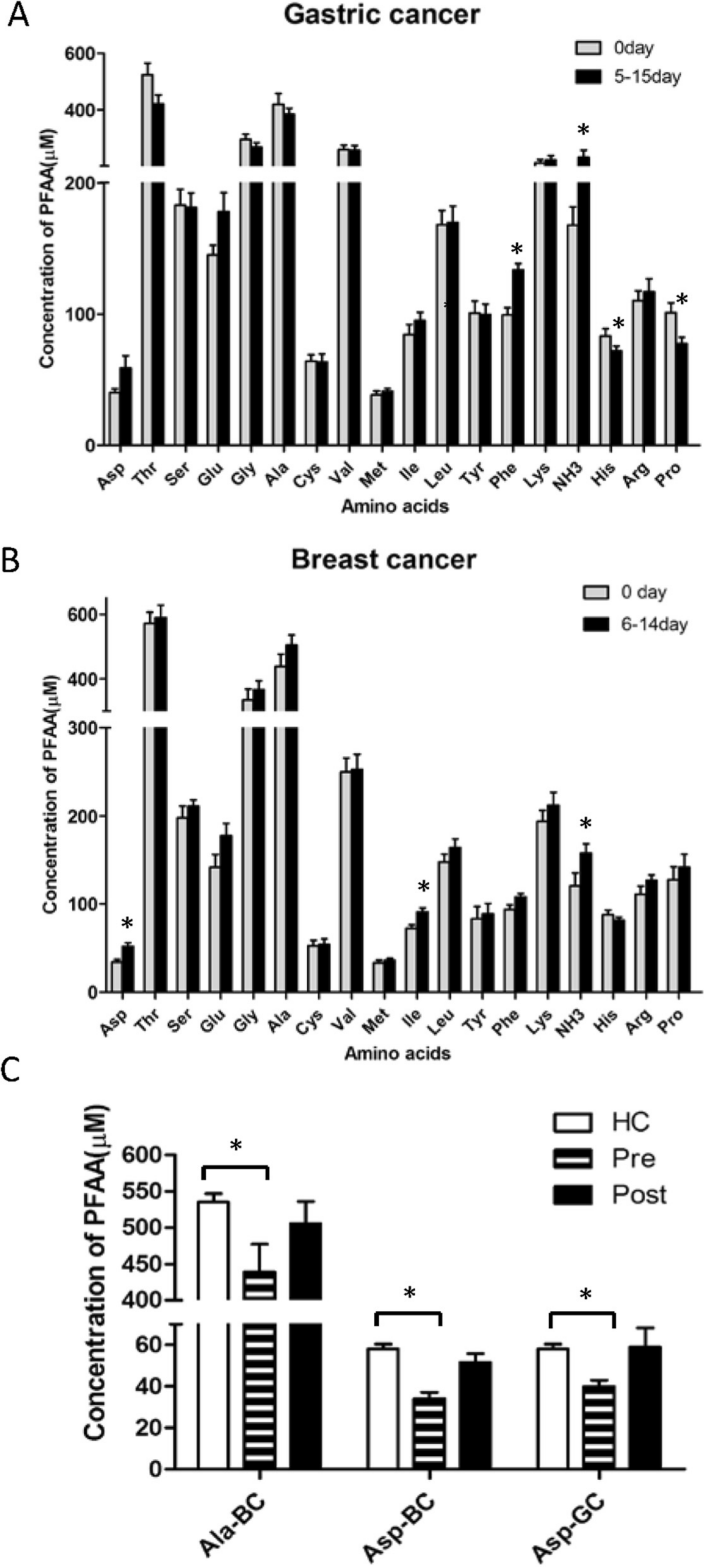
**Dynamic alterations of PFAA profile during perioperative period.** Perioperative alterations of PFAA levels in 15 patients with gastric cancer **(A)** and 10 with breast cancer **(B)** are presented. *P < 0.05, as compared with those on day 0. Amino acids in cancer patients recovered to normal level after tumor removal **(C)**. *P < 0.05 versus HC. HC: healthy control, Pre: preoperation, Post: postoperation, Ala-BC: Ala of breast cancer patients, Asp-BC: Asp of breast cancer patients, Asp-GC: Asp of gastric cancer patients.

To identify perioperative alterations of PFAA profiles, blood samples from 15 GC patients and 10 BC patients were collected before the intervention, and 5–15 days after tumor resection. Levels of His and Pro were significantly decreased and levels of Phe and NH3 were significantly increased in GC patients, 5-15days after surgery (Figure 2A and Additional file 7). Levels of Asp, Ile, and NH_3_ were significantly increased in BC patients, 6–14 days after surgery (Figure 2B and Additional file 7). Level of Asp was significantly increased in GC and BC patients after surgery, and Ala level showed a trend of increase in BC patients after tumor removal (Figure 2C and Additional file 7).

### Correlation between cancer-related PFAAs and clinical features of patients

PFAA levels in BC patients were significantly different from those in HCs, while levels of CA15-3 and CA125 were still normal. In GC group, levels of many tumor markers were higher than those in HCs, such as CA19-9 and CEA (as shown in Additional file 1). Levels of Cys and Ile were significantly correlated with the level of CA19-9, and levels of Glu, Ala, Arg, and Pro were significantly correlated with the level of CEA in GC patients, as shown in Table 3. Level of Asp was correlated with the level of AFP in GC patients. Information on featured pathological parameters, such as estrogen receptor (ER), progesterone receptor (PR), human epidermal growth factor receptor 2 (HER-2), and Ki-67 was also collected in BC patients (Additional file 1) and their correlations with PFAAs were presented in Table 4. Lower levels of Thr, Ser and Gly were significantly correlated with positive ER status, lower level of Gly was significantly correlated with positive PR status and higher level of Ser was significantly correlated with positive HER-2 status. Furthermore, lower level of Ile, Leu, His and BCAA were associated with higher Ki-67 expression in BC patients. Detailed data on correlations between other clinical features of patients with three types of cancers and PFAA levels were shown in the Additional file 2.

**Table 3:** **Correlations between PFAAs and tumor markers of GC patients**

**cphAA**	**AFPr**	**CEAr**	**CA19-9r**
**Asp**	0.29*		
**Glu**		0.31*	
**Ala**		0.29*	
**Cys**			0.29*
**Ile**			0.32*
**Arg**		0.36**	
**Pro**		0.37**	

*and **stand for p-values less than 0.05 and 0.01, respectively.

**Table 4:** **Correlations between PFAAs and pathological parameters of BC patients**

	**ERr**	**PRr**	**HER2r**	**Ki-67r**
**Thr**	−0.46*			
**Ser**	−0.40*		0.50**	
**Gly**	−0.61**	−0.49**		
**Ile**				−0.59*
**Leu**				−0.48*
**His**				−0.62**
**BCAA**				−0.49*

*and **stand for p-values less than 0.05 and 0.01, respectively.

### Effects of selected PFAAs on cell proliferation

**Figure 3:**
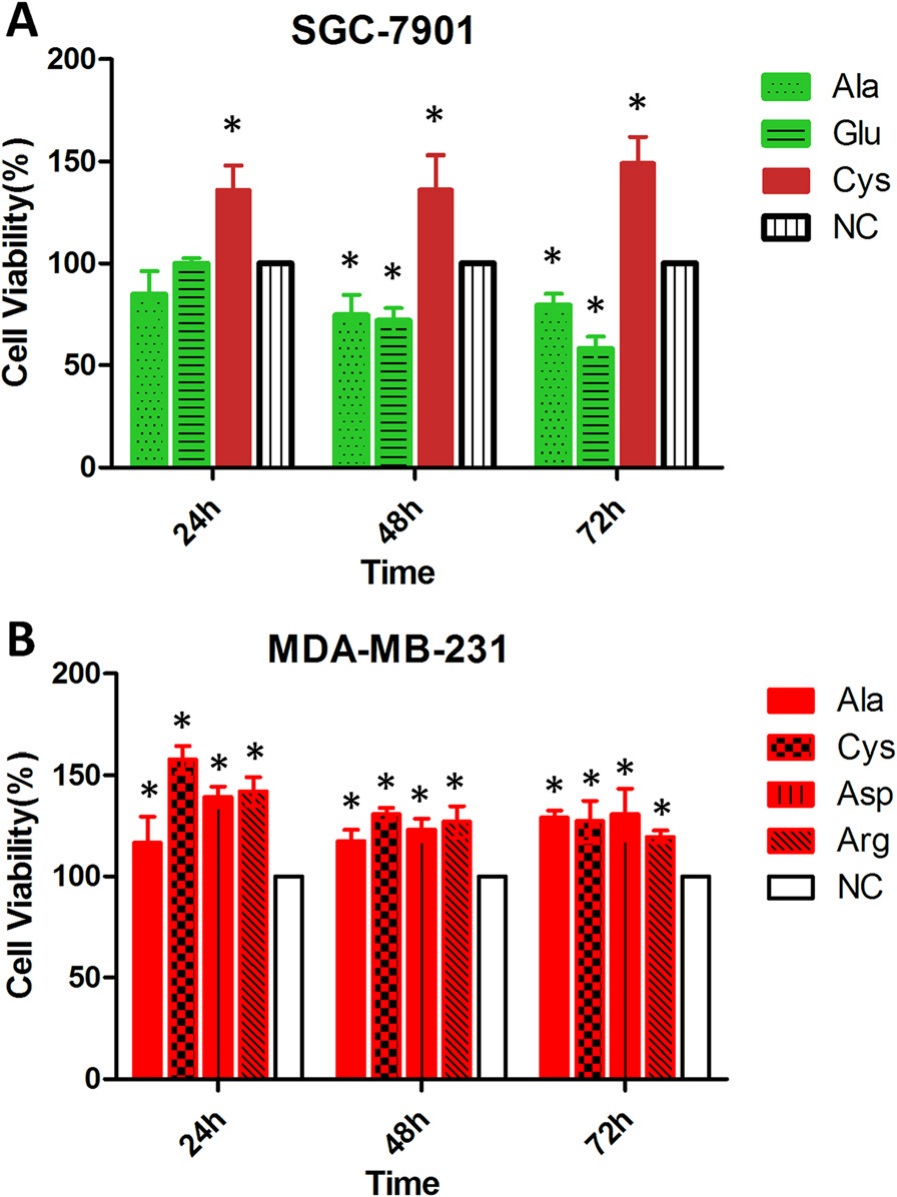
**Effects of different amino acids on cell viability of gastric cancer and breast cancer cells.** Gastric cancer cells SGC-7901 were treated with Ala, Glu, Cys, or control medium (NC) at 10 mM **(A)** and breast cancer cells MDA-MB-231 were treated with Ala, Cys,Asp, Arg or control medium (NC) at 10 mM **(B)**, respectively, for 72 h. Cell viability was determined using CCK-8 proliferation assay. Data were presented as means ± SD of three independent experiments and every experiment was performed in quintuplicate. *P < 0.05 versus NC.

To further investigate the functions of these altered PFAAs identified from patients, 10 amino acids (Ala, Arg, Asp, Cys, Gly, Glu, His, Ser, Thr and Val) were chosen because they had the most obvious alterations in GC and BC patients and significant correlation with patients’ clinicopathological parameters. Their effects on GC cell line SGC-7901 and BC cell line MDA-MB-231 were examined *in vitro*, respectively. According to previous *in vitro* studies on amino acids, we chose concentration of 10 mM for each amino acid [24,25]. We assessed cell viability at 24, 48 and 72 h after amino acid treatments using CCK-8 proliferation assay. Among them, Cys significantly promoted the proliferation of GC cells, while Ala and Glu treatments inhibited cell proliferation (Figure 3A). Ala, Cys, Asp, and Arg treatments could stimulate the proliferation of BC cells (Figure 3B). Other amino acids treatment did not show significant effects on cell viability.

### Effects of alanine on gastric and breast cancer cells

**Figure 4:**
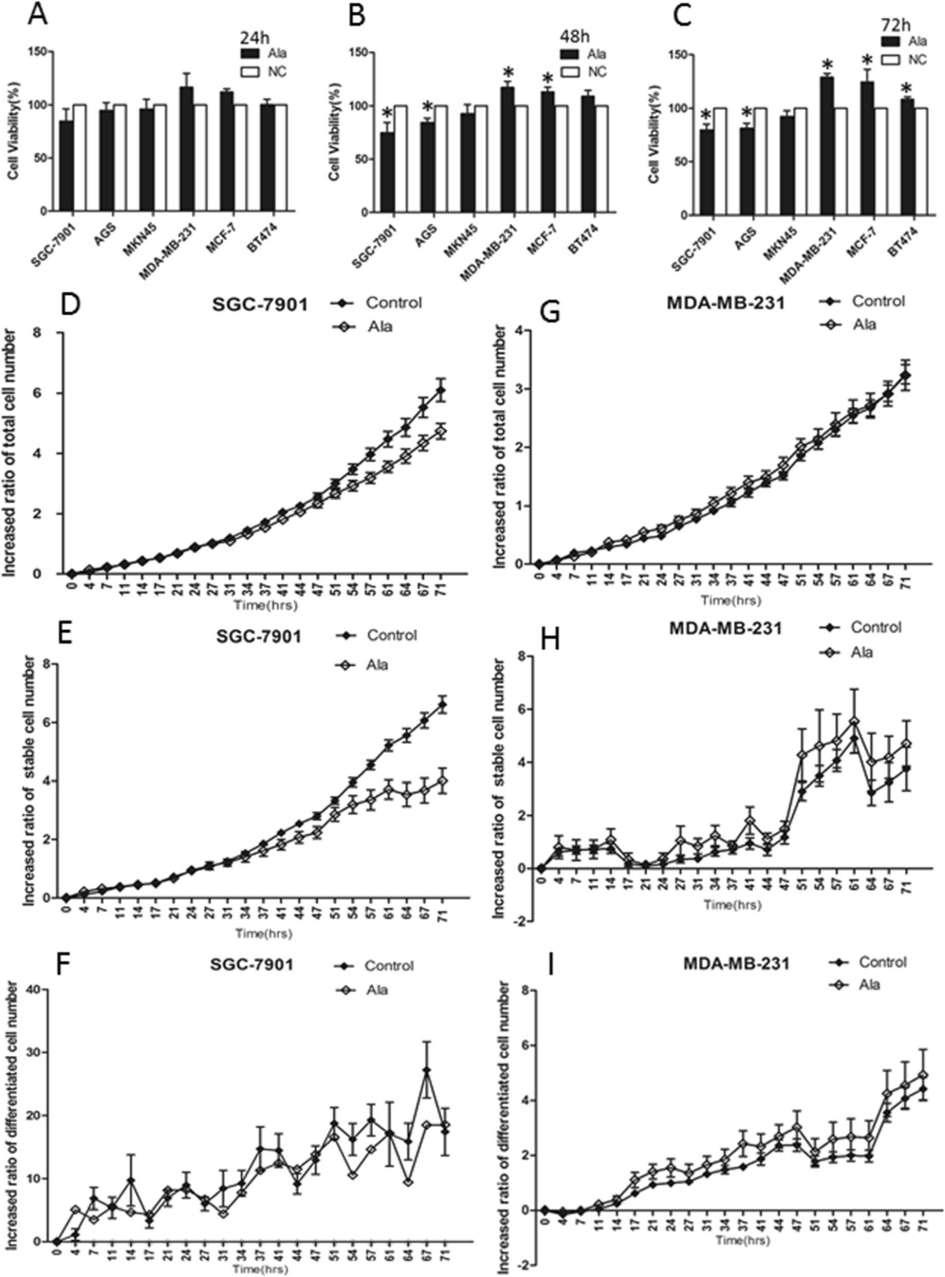
**Biologic functions of Alanine on gastric and breast cancer cell lines** ***in vitro*** **. (A-C)**. Cell viability was assessed by CCK-8 proliferation assay for indicated gastric cancer and breast cancer cells at 24 h **(A)**, 48 h **(B)** and 72 h **(C)** after treatment with 10 mM Ala or negative control medium (NC). **(D-I)**. Dynamic changes of cell numbers were measured by Cell-IQ system. The numbers of total cells **(D)**, stable cells **(E)** and differentiated cells **(F)** from SGC-7901 cell line treated with 10 mM Ala or not. The numbers of total cells **(G)**, stable cells **(H)** and differentiated cells **(I)** from MDA-MB-231 cell line treated with 10 mM Ala or not. Ratios of cell numbers were calculated by formula: [values at indicated time point – values at initial time point]/values at initial time point. Data were presented as means ± SD of three independent experiments. Every CCK-8 assay was performed in quintuplicate and 24 measurements were evaluated in each group for Cell-IQ assay. *P < 0.05 versus NC.

Above results reveal that Ala treatment showed opposite effects on the proliferation of GC cell line SGC-7901 and BC cell line MDA-MB-231, suggesting that Ala may be a key functional amino acid in different cancer metabolisms. The effect of Ala on cell proliferation was further examined in another two GC cell lines (AGS and MKN45) and another two BC cell lines (MCF7 and BT-474), respectively. Accordingly, Ala was found to inhibit proliferation of AGS cells by 16% (48 h treatment) and 19% (72 h treatment), and stimulate proliferation of MCF7 cells by 13% (48 h treatment) and 25% (72 h treatment), and proliferation of BT-474 cells by 8% (72 h treatment), respectively. A mild trend of proliferation inhibition was observed on the effect of Ala on MKN45 cells. Ala treatment inhibited the proliferation of SGC-7901 cells by 21% and increased the proliferation of MDA-MB-231 cells by 29% after 72 h treatment (Figure 4A-C). Because the results from SGC-7901 and MDA-MB-231 cell lines are more significant and promising, we next used these two cell lines to study dynamic alterations of total cell number, proliferation, division, apoptosis and migration after Ala treatment using Cell-IQ system to further explore the potential function of Ala on cancer cells. Ala treatment significantly reduced the numbers of total (Figure 4D) and stable (Figure 4E) GC cells from 54 h and on (p < 0.05), without an effect on BC cells (Figure 4G and H). Ala did not affect the numbers of differentiated GC cells (Figure 4F) or BC cells (Figure 4I).

### Alanine and glutamic acid induced apoptosis of gastric cancer cells

**Figure 5:**
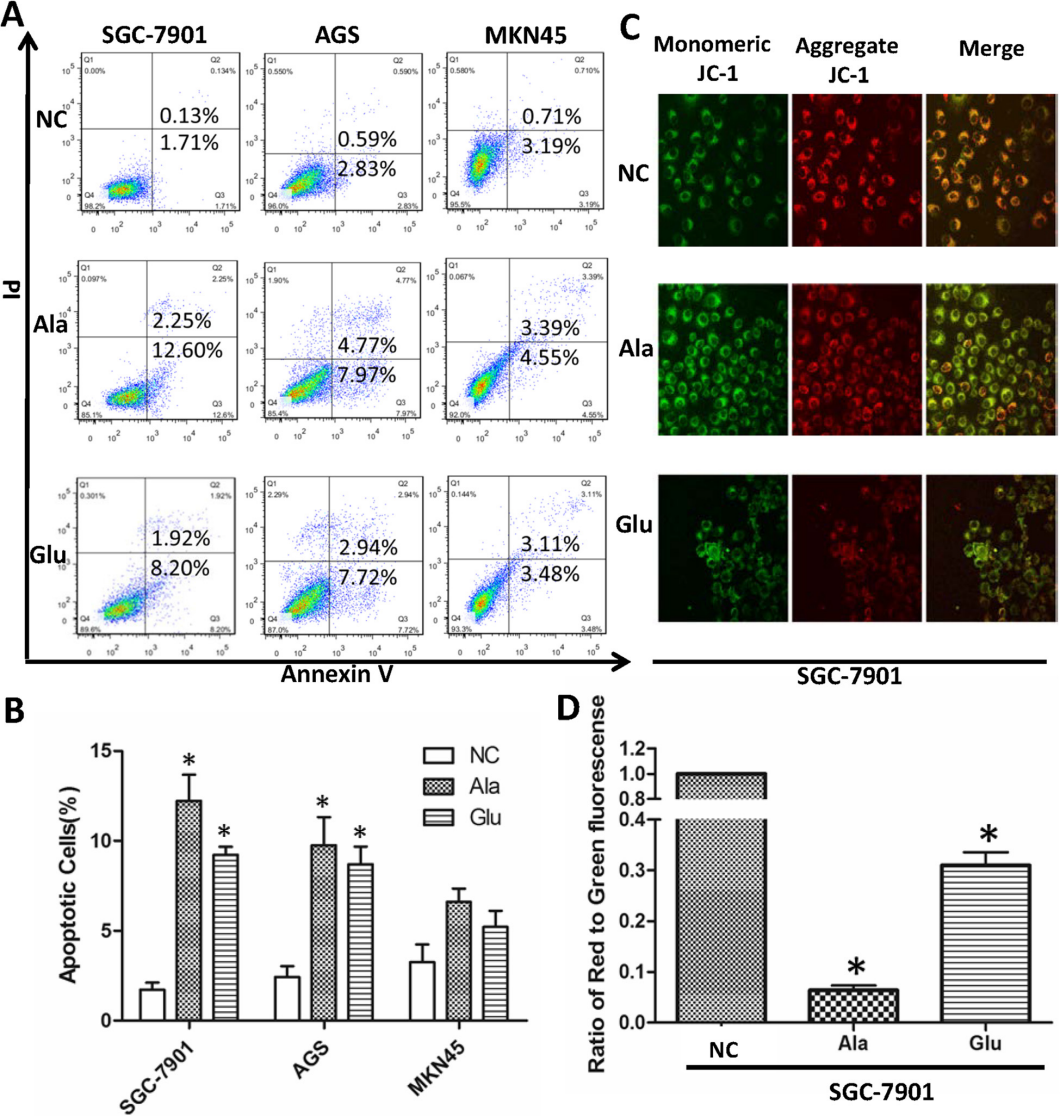
**Alanine and glutamic acid induced apoptosis of gastric cancer cell line. (A-B)**. Induction of apoptosis was detected by flow cytometry with Annexin V-PI staining. Representative flow cytometry plots demonstrating an increase in apoptosis of gastric cancer cell lines after treatment with 10 mM Ala or Glu for 48 h **(A)**. From parallel experiments, apoptosis was quantified **(B)**. **(C-D)**. Induction of apoptosis in SGC-7901 cells through dysregulation of the mitochondrial membrane potential. SGC-7901 cells were treated with 10 mM Ala, 10 mM Glu or negative control (NC) for 24 h, respectively. Then the mitochondrial membrane depolarization of cells was examined by JC-1 dye staining with confocal microscopy. Left and middle images showed green JC-1 monomer and red JC-1 aggregate, respectively. The right images showed the overlay of two images. Green fluorescence indicates the presence of depolarized mitochondria (apoptotic cells). Red fluorescence indicates normally functional and polarized mitochondria (healthy cells) **(C)**. From parallel experiments, quantitative assessment of mitochondrial membrane depolarization in SGC-7901 cells was presented as ratio of JC-1 aggregates/monomers **(D)**. Data were represented as mean ± SD of three independent experiments. *P < 0.05 versus NC.

Since Ala and Glu treatments inhibited proliferation of GC cells (Figure 3A), we next examined apoptosis of three GC cell lines SGC-7901, AGS and MKN45 after Ala or Glu treatment by flow cytometry. Apoptosis of SGC-7901 and AGS cells were increased after treatment with 10 mM Ala or Glu for 48 h (Figure 5A,B). Furthermore, we used SGC-7901 cells to assess the effect of Ala on mitochondrial membrane potential (MMP) assay. Mitochondria furnishes cellular energy through respiration and regulates cellular metabolism to maintain the growth, differentiation and proliferation of cells [26]. During apoptosis, MMP is depolarized, as an indicator of mitochondrial dysfunction [26] and this can be detected by JC-1 dye. In healthy cells, JC-1 accumulates in the mitochondria with aggregated, red fluorescence, while in apoptotic cells with decreased MMP, JC-1 is diffused in the cytosol as green fluorescent monomers. Therefore a decrease in the red/green fluorescence intensity ratio of JC-1 staining indicates mitochondrial depolarization and apoptosis. MMP was depolarized in GC cells after the treatment of Ala or Glu at 10 mM for 24 h (Figure 5C,D).

## Discussion

The plasma metabolic profiles have been investigated in a variety of human cancers [27,28]. Our results suggested that different tumor origins may lead to disease-specific PFAA profile. Alterations of PFAA profiles were closely correlated with clinical features of patients such as molecular tumor markers in GC and hormone receptors and Ki-67 expression in BC. In particular, concentrations of Thr, His, EAAs, and GAAs were significantly correlated with lymph node metastases in patients with GC, indicating that specific PFAAs may act as biological indicators for the metastatic potential of GC. We further found that levels of selected tumor-related metabolites in cancer patients (such as Asp, Ala, His, Pro, Phe, Ile, and NH3) dramatically changed 5–15 days after surgery. *In vitro*, we examined cytological effects of 10 tumor-related amino acids that were identified from PFAA profiles on gastric and breast cancer cells. Among them, we found that Ala and Glu inhibited the proliferation of GC cells, while Ala promoted proliferation of BC cells. The opposite effects of Ala on GC and BC cell indicate the cancer specific role of Ala.

Although evidence of relationships between PFAA profiles and certain types of cancer was introduced early, only a few studies have investigated the use of PFAA profiles for diagnosis. Recently Shingyoji *et al.* observed high reproducibility of the discriminating performance for patients with lung cancer compared to previously reported results. Their results also show that combinational application of PFAA profiles and tumor markers may even improve the clinical utility of tumor markers [29]. A Japanese research collecting samples from approximately 200 patients with lung, gastric, colorectal, breast, or prostate cancer revealed significant differences in the PFAA profiles between the controls and the patients with any of the five types of cancer listed above, regardless of cancer stage [18]. Similar findings were observed in patients with renal cell carcinoma [30] and colorectal cancer [31]. These findings suggest that PFAA profiling has great potential for improving cancer screening and diagnosis and for understanding disease pathogenesis. In addition, other studies have revealed correlation between PFAA profiles and clinicopathological characteristics such as pathological grading, lymph node metastasis and clinical stage of patients with esophageal squamous cell carcinoma [16] and cervical cancer [32]. The longitudinal studies with larger sample sizes and longer follow up are needed to further justify whether PFAA might predict prognosis.

Previous reports have shown that ‘cachexia’ is an important cause of PFAA alterations of cancer patients, which is a condition where patients lose body mass that cannot be reversed nutritionally during the disease course [33]. Cancer patients with cachexia had different amino acid patterns from malnourished patients for other reasons [34]. There is no sign of cachexia in our subjects, as most of them did not lost weight or have decreased serum albumin level. Additionally, significant alterations in PFAA profiles were not observed in GC patients between early stage and advanced stage. Thus, it is reasonable to speculate that the identified changes of PFAA profiles do not result from poor nutrition caused by tumor progression. Significant perturbations of PFAA patterns in patients with cancer are usually caused by dysfunctions of host metabolism [12]. Our study demonstrated that GC patients had the most distinctive alterations since the majority of PFAA levels seemed to decrease significantly. The difference from other cancers may be related to absorption ability of the gastrointestinal tract and hepatic metabolism in gastric cancer patients [12]. Another proposed mechanism might be the loss of protein-rich exudates into gastrointestinal tract from ulcerative cancer masses [12]. The PFAA profile of BC patients is generally close to healthy group among three types of cancer, which is in concordance with previously reports [35]. This is probably because the growth rate and aggressiveness of BC are lower than those of other metabolically active cancers [36]. Notably, PFAAs pattern in TC patients was greatly changed and this might be caused by upstream genomic changes [37]. To our knowledge, this is the first study reporting the PFAA profile of TC patients.

Lymph node metastasis is one of the most important prognostic factors and provides important information for accurate disease staging and appropriate treatments for GC patients. However, preoperative diagnosis of lymph node metastases has remained unsatisfactory [38]. Our results showed that Thr, His, EAAs, and GAAs were significantly lower in lymph node positive GC patients compared with negative ones. Since Thr and His were included in GAAs, the alteration might be due to increased gluconeogenesis in GC with higher metastatic potential. The decreased Thr level was also significantly associated with larger tumor size (>5 cm) and deeper tumor invasion (>T3), which suggests the important role of Thr in GC development. Thr has been reported to decrease in many cancers including GC [12,18]. Thr can be converted to pyruvate, which is located at a key intersection of metabolic network and has direct links to a number of other amino acids [39,40]. However, larger sample size is required to verify these observations.

PFAA profiling provides an instantaneous metabolic snapshot of the human body [19]. It may be an effective tool to monitor treatment response of patients. A previous study has shown that characteristic plasma amino acid patterns of rats with tumors were reversed by tumor removal [41]. Hence, we were inspired to analyze the dynamic metabolism of perioperative cancer patients by PFAA profiling. After tumors were surgically removed, preoperative deficiencies of Asp restored to normal levels in both BC and GC patients. Similar alteration trend was observed for Ala of BC patients. These preoperative deficiencies of amino acids may be caused by increased uptake and utilization of amino acids by tumor cells. PFAAs shall be monitored until tumor recurrence and metastasis. If these PFAAs drop again with tumor progression, it verifies clinical significance of tumor-related amino acids. Translocation or redistribution of PFAAs in cancer patients to support visceral or tumor protein synthesis is believed to be crucial. An unnatural PFAA pattern might be shown through the total reflection of cancer-induced protein metabolism in tumors, the liver and skeletal muscle in cancer patients [12]. A study from France reported that surgical tumor removal induced a normalization of aminoacidemia [42]. However, the effect of surgery and wound healing process after surgical trauma on amino acid metabolism and PFAA profile is still unclear. An animal study has shown that pulmonary blast injury induces prompt arginine elevation through NO synthase [43]. Moreover, a persistent drop of arginine, which contributes to T cell dysfunction and decrease of nitric oxide (NO) production, was found to significantly increase susceptibility to infections and organ failure after trauma or surgery. Dietary therapy containing arginine at supra-physiologic levels along with other components is related to improvements in T cell function, NO production, and a significant decrease in infection rates [44]. Therefore, deeper understanding of perioperative dynamic PFAA profile is also important for better recovery from surgery.

Genomics and proteomics are used to identify many blood-based biomarkers of GC and BC. A small genomic and proteomic changes can be amplified multiple times at the metabolite level and quantitatively measured [45]. In our study, most of BC and TC subjects are defined at relative early stages, and their clinical blood samples revealed no anomalies. However their PFAA profiles were significantly different from the control group. In the GC patients’ group, some PFAAs levels were correlated with typical tumor markers such as CEA and CA19-9. These results suggested that tumor-specific amino acids identified by PFAA profiling may be potential accomplishment for early cancer detection. In addition, PFAA profile was correlated with expression of ER, PR, HER2 and Ki-67 in BC patients, which are critical for treatment choices and clinical outcomes [46,47]. Some AAs were associated with negative prognostic markers as Ki-67 but others with hormone factors which implies good prognosis. These results indicate biological function of each amino acid varies during tumor progression. This might also be due to the upstream genomic and proteomic change and provide potential classification information before surgery.

To further study the mechanism of cancer-related PFAAs, we found Ala and Glu were pro-apoptotic GC cells as evidenced by flow cytometry and MMP assay. These data are also consistent with data obtained from CCK-8 and Cell-IQ assays. Previous studies have reported that Ala and Glu were significantly up-regulated in apoptotic HepG2 and HEK293 cells and they may be relevant signature molecules of apoptosis. The increase of Ala and Glu in apoptotic cells was explained by their association with taurine metabolism [48]. However, Ala showed opposite effects on BC cells by promoting cells proliferation, which indicates the cancer type-specific role of Ala. Ala is the key protein-derived glucose precursor used by the liver [49]. The decrease of plasma Ala concentration in the GC group supports the notion that tumor malignancy is associated with an increase in gluconeogenesis [50]. Ala was found to be protective against acute liver failure and melanoma [24,51]. As for breast cancer, elevation of Ala concentrations may enhance gluconeogenesis and promote proliferation of breast cancer cells *in vitro*. However, no significant alteration of plasma Ala level was seen in BC patients group. Studies with large sample volume are needed to further explore the tumor type-specific role of Ala in different types of cancer. It should also be noted that the tumor microenvironment made up of various immune and stroma cells influence amino acid metabolisms in tumor cells. Poschke and colleagues discovered tumor-dependent impact of breast cancer on serum AA levels, and positive correlation of certain amino acid levels and pro-inflammatory immunological factors and a more aggressive intrinsic tumor subtype [52]. Our *in vitro* findings should be further investigated in animal models or human tumor samples.

Since PFAAs are biosynthesized in an interrelated fashion, the PFAA profile is a complex network containing rich amount of information. For instance, Ala and Pro are biosynthesized from Glu via aminotransferase [53]. Tumor growth requires Glu, Gly, Asp and ammonia for purine and pyrimidine synthesis, which brings a selective demand on these metabolites [54]. Actually, our data showed that the levels of these amino acids in patients with different cancers were lower than those of healthy individuals. Arg has long been recognized as an essential nutrient for tissue healing and a critical component of immunonutrition [55]. More importantly, there is evidence suggesting that Arg stimulates tumor growth [56]. We found Arg levels raised in all patient subjects. We also found that plasma level of BCAAs in GC patients was significantly lower than that of healthy subjects. BCAAs, oxidized peripherally, serve as a source of fuel to decrease protein degradation and stimulate protein synthesis in liver and muscle [57]. Circulating level of BCAAs tends to decrease in patients with advanced liver cirrhosis [58]. Our results may indicate disrupted liver function in GC patients.

Unlike transcripts or proteins with multiple modifications, each amino acid is uniquely stable and the entire amino acids family is relatively small in humans. In addition, PFAA alterations may amplify the upstream fine tuning. From our results, we demonstrated that PFAA profiling might be developed as a promising diagnostic method for cancer patients. However, additional large-scale studies are required to fully evaluate clinical utility of this profile. Furthermore, integrating novel discovery of clinical and laboratory study provides better understanding on biological function of cancer type-specific amino acids for gastric cancer and breast cancer. The integrative analysis of clinical informatics and novel biomarkers may generate more meaningful studies in translational medicine [59,60].

## Conclusions

PFAA patterns of cancer patients are dynamically altered during the perioperative period. Tumor-related amino acids identified by dynamic study of PFAA pattern might be promising biomarkers for diagnosis of cancer patients. Their biological effects on cancer cell proliferation are worth further evaluation for metabolic treatment in the future.

## Additional files

Additional file 1:
**Clinical information of cancer patients.**
Additional file 2:
**Correlations between PFAAs and clinical information in three kinds of cancer patients.**
Additional file 3:
**PFAA profiles of patients with different cancers and healthy controls.**
Additional file 4:
**PFAA profiles of GC patients at stage 0-III and stages IV.**
Additional file 5:
**PFAA profiles of patients with GC, BC or TC at stage 0-III and healthy controls.**
Additional file 6:
**Association of PFAA profiles with GC clinicopathological characteristics.**
Additional file 7:
**Perioperative alterations of PFAAs in gastric and breast cancer patients.**

## Abbreviations

PFAA: Plasma free amino acid
GC: Gastric cancer
BC: Breast cancer
TC: Thyroid cancer
HC: Healthy control
CCK-8 assay: Cell counting kit-8 assay
MMP: Mitochondrial membrane potential
Ala: Alanine
Arg: Arginine
Asp: Aspartate
Cys: Cysteine
Glu: Glutamic acid
Gly: Glycine
His: Histidine
Ile: Isoleucine
Leu: Leucine
Lys: Lysine
Met: Methionine
NH3: Ammonia
Phe: Phenylalanine
Pro: Proline
Ser: Serine
Thr: Threonine
Tyr: Tyrosine
Val: Valine
NEAA: Non-essential amino acids
EAA: Essential amino acid
BCAA: Branched-chain amino acid
GAA: Glucogenic amino acid
TAA: Total amino acid
CEA: Carcinoembryonic antigen
CA19-9: Carbohydrate antigen19-9
CA125: Carbohydrate antigen 125
CA15-3: Carbohydrate antigen 15–3
AFP: Alpha-fetoprotein
ER: Estrogen receptor
PR: Progesterone receptor
HER-2: Human epidermal growth factor receptor 2
